# Temporary worsening of mitral regurgitation due to conduction disturbance after transcatheter aortic valve implantation

**DOI:** 10.1186/s40981-021-00491-3

**Published:** 2021-12-18

**Authors:** Takeyuki Sajima, Taichi Onimaru, Shigehito Sawamura

**Affiliations:** Department of Anesthesia, Teikyo University, 2-11-1 Kaga, Itabashi-ku, Tokyo, 173-8605 Japan

**Keywords:** Conduction disturbance, Dyssynchrony, Mitral regurgitation, TAVI

## Abstract

**Background:**

Mitral regurgitation after transcatheter aortic valve implantation (TAVI) can be caused by various etiologies.

**Case presentation:**

An 81-year-old woman with mild mitral regurgitation and complete right bundle branch block was scheduled to undergo TAVI under general anesthesia. After the deployment of the prosthetic valve, electrocardiography depicted a wide QRS wave and bradycardia, suggestive of complete atrioventricular block. Although there was no lesion indicative of tissue injury to the valve itself, worsening of mitral regurgitation was identified on transesophageal echocardiography. The hemodynamic condition was stable, and no additional procedure was performed. Electrocardiography depicted a return to a narrow QRS wave 3 days after surgery, and the mitral regurgitation appeared comparable to the preoperative grade. We assumed that the worsening of mitral regurgitation was caused by dyssynchrony in the left ventricle due to the conduction disorder.

**Conclusions:**

Mitral regurgitation after TAVI needs observation, including the determination of the etiology and treatment principle.

## Background

The frequency of transcatheter aortic valve implantation (TAVI) for aortic valve stenosis has increased owing to its indications in patients with a myriad of cardiac complications that may affect the prognosis. Preoperative mitral regurgitation or postoperative severe mitral regurgitation is a known factor associated with the worsening of prognosis. Herein, we report a case of an altered severity of mitral regurgitation resulting from a change in left ventricular conduction after Evolute-PRO® (Medtronic, Irvine CA) implantation.

## Case presentation

**Fig. 1:**
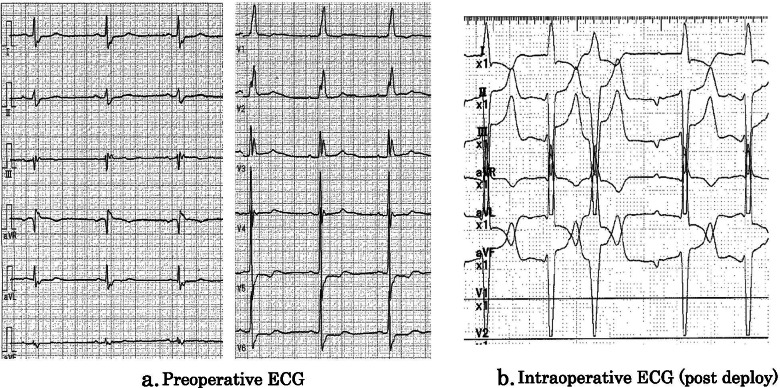
Preoperative and intraoperative ECG (post-deploy). **a** ECG showed regular sinus rhythm with complete right bundle branch block. **b** ECG showed complete atria-ventricular block, with left bundle branch block

**Fig. 2:**
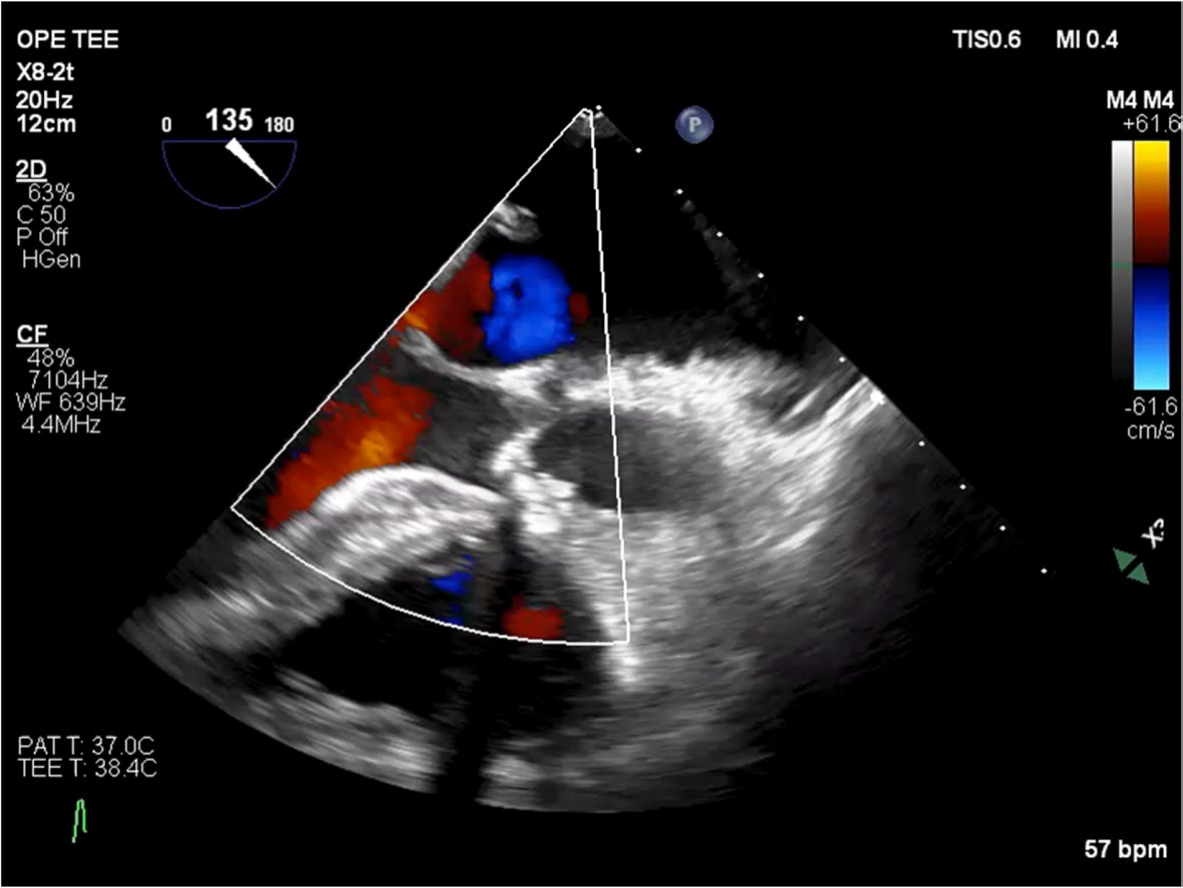
Intraoperative transesophageal echocardiography (TEE) (pre-deploy). Mid-esophageal long-axis view. Preprocedural examination shows trivial mitral regurgitation. The aortic valve was highly calcified, and transaortic valve color flow Doppler ultrasound shows a mosaic pattern of blood flow

**Fig. 3:**
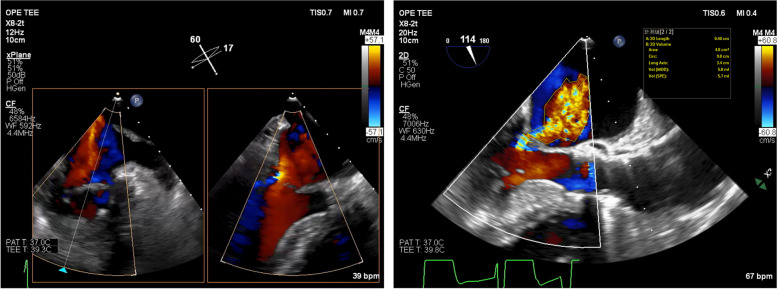
Intraoperative transesophageal echocardiography (TEE) (post-deploy). Movie. In systolic period, central regurgitation flow was detected in indentation of mitral valve with color Doppler mode. Left side view showed mid-commissural view. Regurgitant flow of different time phases are shown. LV long axis view. Vena contracta was 4.0mm. This semi-quantification revealed mild to moderate regurgitation

**Fig. 4:**
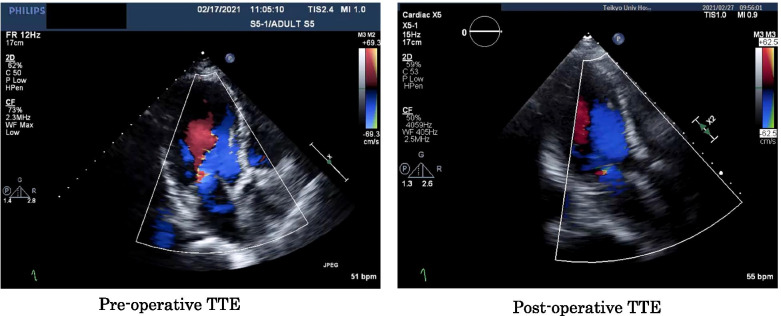
Pre- and postoperative transthoracic echocardiography (TTE). Comparison of post-operative TTE and that of pre-operation. Mitral regurgitation was relieved

An 81-year-old female patient with severe aortic stenosis was scheduled to undergo TAVI under general anesthesia. She had a medical history of well controlled type1 diabetes. The prosthetic valve used in this case was 23mm Evolute PRO ® (Medtronic, Irvine CA). Preoperative transthoracic echocardiography (TTE) revealed ejection fraction of 68%, no asynergy, maximum pressure gradient of 104 mmHg, mean pressure gradient of 56 mmHg through the aortic valve orifice, valve area index of 0.67 cm^2^/m^2^, and extremely severe aortic valve stenosis. Preoperative electrocardiography (ECG) revealed regular sinus rhythm with complete right bundle branch block (Fig.1a). The preoperative enhanced computed tomography revealed a ring circumference of 61.1 cm, area of 285.1 cm^2^. Transfemoral artery TAVI was performed under general anesthesia using midazolam 2 mg, fentanyl 100 mcg, and rocuronium 50 mg before intubation. Anesthesia was maintained intraoperatively using sevoflurane and remifentanil. After the induction of anesthesia, a highly calcified valve leaflet and a mild mitral regurgitation was observed in the screening TEE (Fig. 2). The pre-dilation was first performed using the balloon. After the pre-dilation, the prosthetic valve was deployed under controlled pacing (heart rate: 120 beats/s). The depth of implantation was difficult to determine. Deployment failed twice due to occurrence of complete atria-ventricular block and succeeded at the third attempt. Finally, the valve was placed just below the aortic annulus, but ECG showed wide QRS wave, which was indicative of a left bundle branch block with a complete atrioventricular block (Fig. 1b). Although no dysfunction was observed in the prosthetic aortic valve or left ventricular systolic function, the mitral regurgitation deteriorated compared to the preoperative TTE grade. Central regurgitation flow was detected in the mitral valve from the systolic period, but no tendinous rupture or valve injury was observed on the intraoperative transesophageal echocardiography (TEE) (Fig. 3). Vena contracta was 4.0mm that indicated mild to moderate mitral regurgitation (Fig. 3). A pacemaker was not used because the hemodynamic condition of the patient was stable after surgery. For a while, the ECG and mitral regurgitation did not show any change; however, 3 days after the procedure, the ECG showed a return to sinus rhythm and the mitral regurgitation returned to its preoperative state (Fig. 4) without any complications.

## Discussion

The persistence of moderate or severe mitral regurgitation after TAVI is associated with worsening of prognosis and is, therefore, a highly critical issue [1]. Usually, its severity does not increase after the surgical management of aortic stenosis because of the reduction in the left ventricular afterload [2]. Most factors responsible for the worsening mitral regurgitation post-TAVI were structural causes such as ruptured chordae tendineae or mitral valvular damage caused by a stiff surgical guide wire, or functional causes such as systolic anterior motion, temporary right ventricular pacing, and bundle branch block such as left ventricular conduction disturbance caused by valve implantation [3, 4].

The bioprosthetic valve used in this patient, i.e., Evolute PRO ® (Medtronic, Irvine CA), is a self-expanding valve, which is associated with a greater possibility of postoperative left bundle branch block or permanent pacemaker implantation due to complete atrioventricular blockade compared to a balloon-expandable valve. Left ventricular conduction disturbance causes left ventricular dyssynchronic contraction, which in turn may result in functional mitral regurgitation [3]. The causes of the post-TAVI conduction disturbance are usually surgical factors, such as incorrect positioning or oversizing of the prosthetic valve. In addition to these, intraoperative rapid and controlled right ventricular pacing itself may cause left ventricle conduction disturbance, and the mitral regurgitation may worsen if backup pacing is used post-TAVI. Conversely, mitral regurgitation in backup pacing or temporary left ventricular conduction disturbance may decrease with the normalization of conduction.

In this case, the patient’s heart rate was approximately 40 beats/min and ECG revealed complete atria ventricular block and a wide QRS wave pattern suggestive of a left bundle branch block after deploying the valve. A pacemaker was not used because the patient’s hemodynamic condition was stable, and this abnormal electronic conduction was thought to be temporary. Because most conduction disturbances recovered in about 24h, it seemed to be appropriate to wait for about 48 h in this case [5]. If conduction disturbance persists for 24–48 h after TAVI or appears later, we should consider using a permanent pacemaker or resynchronization therapy [6].

Previous studies reported the appearance of a left ventricle dyssynchrony in the event of a left ventricular conduction disturbance [3], and its disappearance with re-synchronized therapy improved mitral regurgitation [7]. In this patient, we suspected that temporary worsening of the mitral regurgitation was induced by dyssynchrony caused by left ventricular conduction disturbance due to direct or indirect injury to the conducting tissues, because surgically induced mitral valve injury was not detected on intraoperative TEE and fluoroscopy. Although conduction disturbance in the left ventricle was observed on ECG, unfortunately we could not show left ventricular dyssynchrony because measurements were not performed on intraoperative TEE and post-operative TTE.

Previous reports have shown that risk factors for developing complete atrioventricular block or left bundle branch block after TAVI were preoperative conduction disturbance, female, a history of CABG, diabetes, depth of implantation, and severe aortic valve calcification. This case was a high-risk case of conduction disturbance because she had risk factors of CRBBB, female, diabetes, and severe aortic calcification [5].

The timing of the onset and duration of post-procedural conduction disturbance is uncertain: it is not clear whether it occurs early or later and if it is permanent or temporary [5].

Although conduction disturbance occurred immediately after deployment in our case, tardive post-procedural conduction disturbance may occur in self-expanding valves, and it is possible that its influence on cardiac function becomes apparent after TAVI. We should pay attention if mitral regurgitation exists preoperatively because of the possibility of a worsening regurgitant flow due to conduction disturbance.

Postoperative mitral regurgitation after TAVI is a very noteworthy complication and is reportedly associated with poor prognosis [1]. We encountered a case in which a temporary worsening of mitral regurgitation was observed after the TAVI procedure. This was attributed to the left ventricular conduction disturbance, which may be temporary. As far as we know, there has been no case report describing deterioration of mitral regurgitation induced by conduction disturbance during TAVI. We should know mitral regurgitation after the TAVI procedure caused by dyssynchrony is considered to improve with restored conduction.

## Data Availability

Not applicable

## Abbreviations

TAVI: Transcatheter aortic valve implantation
TEE: Transesophageal echocardiography
TTE: Transthoracic echocardiography
ECG: Electrocardiography
CRBBB: Complete right bundle branch block
